# A Symmetric Plaintext-Related Color Image Encryption System Based on Bit Permutation

**DOI:** 10.3390/e20040282

**Published:** 2018-04-13

**Authors:** Shuting Cai, Linqing Huang, Xuesong Chen, Xiaoming Xiong

**Affiliations:** 1 School of Automation, Guangdong University of Technology, Guangzhou 510006, China; 2 School of Applied Mathematics, Guangdong University of Technology, Guangzhou 510006, China

**Keywords:** bit-level permutation, image encryption, PRRABPM, plaintext related

## Abstract

Recently, a variety of chaos-based image encryption algorithms adopting the traditional permutation-diffusion structure have been suggested. Most of these algorithms cannot resist the powerful chosen-plaintext attack and chosen-ciphertext attack efficiently for less sensitivity to plain-image. This paper presents a symmetric color image encryption system based on plaintext-related random access bit-permutation mechanism (PRRABPM). In the proposed scheme, a new random access bit-permutation mechanism is used to shuffle 3D bit matrix transformed from an original color image, making the RGB components of the color image interact with each other. Furthermore, the key streams used in random access bit-permutation mechanism operation are extremely dependent on plain image in an ingenious way. Therefore, the encryption system is sensitive to tiny differences in key and original images, which means that it can efficiently resist chosen-plaintext attack and chosen-ciphertext attack. In the diffusion stage, the previous encrypted pixel is used to encrypt the current pixel. The simulation results show that even though the permutation-diffusion operation in our encryption scheme is performed only one time, the proposed algorithm has favorable security performance. Considering real-time applications, the encryption speed can be further improved.

## 1. Introduction

With the dramatic development of Internet technology, a great deal of sensitive information conveyed by digital images has been transmitted over public networks. The security problems of image transmission have become increasingly serious, especially for those related to confidential, medical, military, or commercial affairs. However, since digital images have some inherent characteristics (e.g., high redundancy, large data capacity, and strong correlation between adjacent pixels), the traditional block ciphers like Data Encryption Standard (DES), International Data Encryption Algorithm (IDEA), Advanced Encryption Standard (AES), RSA (Rivest–Shamir–Adleman), etc. do not have high performance. In recent years, chaotic maps have been used in image encryption, which have benefited from their excellent properties, such as strong ergodicity as well as sensitivity to initial conditions and control parameters. As early as 1989, Matthew suggested the use of logistic maps to generate pseudo-random numbers, which can be used to encrypt messages [1]. In 1998, a new symmetric block encryption scheme proposed by Fridrich [2] drew a great deal of attention. The architecture is similar to the one Shannon introduced in [3], which includes a pixel-level permutation-diffusion structure. In the permutation stage, the pixel position is scrambled to disturb the strong correlation between two adjacent pixels of the original image, but the pixel’s statistical property is not changed. Later, in the diffusion stage, the pixel values are modified to achieve a uniform distribution of pixel values [4,5,6,7,8,9]. For instance, Gao et al. used a total shuffling matrix to change the image pixel positions in the permutation stage, and then a hyper-chaotic system is used to modify the pixel values of the shuffled-image to obtain the cipher-image [4]. In [5], four values obtained from the logistic map are used to disorder four equal sub-images divided from the plain-image, and then a total shuffling matrix is used to shuffle the position of the pixels in the whole plain-image. Finally, the four sub-images are diffused simultaneously in parallel. More recently, chaotic systems have been used to encrypt images in specific fields. For example, Abundiz-Pérez et al. proposed a high-security and fast fingerprint image encryption scheme based on hyperchaotic Rössler maps [6]. In [8], a novel symmetric encryption algorithm based on confusion-diffusion architecture is provided and used to encrypt clinical information. All simulation results of the encryption system show its effectiveness, security, and robustness. Compared with gray-level images, color images can provide more information, so color image encryption attracts increasing attention. In recent years, plenty of chaos-based color image encryption algorithms have been proposed [10,11,12,13,14,15,16,17]. In order to make the RGB components of a color image affect each other and obtain high security, Wang et al. [10] used a chaotic system to encrypt these three components at the same time. In [12], Wang et al. transformed the R, G, and B components of a color plain-image into a matrix. When the matrix is passed through a permutation operation using zigzag path scrambling and a substitution process, the ciphered color image is obtained. Later, in 2017, Huang et al. [15] used Logistic map to diffuse the color image, then the RGB components are scrambled by Logistic mapping. Secondly, double random-phase encoding is used to encrypt the three scrambled sub-images into one encrypted image.

A bit-level permutation (BLP)-based cryptosystem has been proposed as a new image encryption algorithm [18,19,20,21,22,23,24,25,26]. BLP considers images as 3D bit matrices (width, height, and bit-length). So, the basic operation unit in the permutation stage is performed on bits rather than pixels. As the bits in different bit-planes of an image contribute different effects to visualization, Xiang et al. [18] proposed an image encryption scheme in which only the higher four bits of each pixel are encrypted and the lower four bits are unchanged. Compared with pixel-level permutation, bit-level permutation not only changes the position of the pixel, but also modifies its value. Although several rounds of 2D scrambling on each bit-plane of a plain-image are performed in some bit-level-based image encryption algorithms, the statistical property of each scrambled bit-plane are not changed. However, by combining these scrambled bit-planes to produce encrypted pictures, the statistical property of pixels in the encrypted image will be changed. For example, Zhu et al. permuted the higher four bit-planes independently and permuted the four lower bit-planes together with the Arnold cat map in [19]. Due to the problem that the permutation using 2D chaotic maps has a repeated pattern and there are strong correlations among the adjacent bit-planes (especially between higher bit-planes like the seventh and the eighth bit-planes) [21], the BLP algorithm should allow one bit in any plane to be moved to any other position in any plane. Recently, various schemes with improved properties have been proposed [22,23,24,25,26]. In [23], a symmetric chaos-based image cipher with a spatial bit-level permutation strategy is proposed. Compared with the recently proposed bit-level permutation methods, the confusion and diffusion effect of this new method is superior, as the bits are shuffled among different bit-planes rather than within the same bit-plane. Zhang et al. [26] proposed a new 3D bit matrix permutation mechanism which can access the bits of the plain-image randomly rather than in an orderly fashion. Furthermore, for color image encryption, bit-level permutation-based encryption algorithms have the advantages that they can achieve the interaction between RGB components in the scrambling phase, which can improve the security of encryption.

However, for most chaotic-based image encryption schemes, the relationship between permutation stage, diffusion stage, and the plaintext image is independent. Such algorithms have the following security flaws: (1) the architecture is insensitive to the original image; (2) the statistical property of the original image can be observed once the diffusion key or diffusion sequence is cracked; (3) the algorithm cannot resist chosen-plaintext and chosen-ciphertext attack. As shown in Table 1, most of the permutation-diffusion structure-based cryptosystems [27,28,29,30,31,32,33] are attacked by chosen-plaintext attack and chosen-ciphertext attack.

More recently, in order to resist the powerful chosen-plaintext and chosen-ciphertext attacks, a plaintext related image encryption scheme was proposed [12,23,34,35,36,37,38,39,40,41]. For some algorithms, the previous encrypted pixel is used to encrypt the current pixel, and after several rounds of processing in the diffusion stage of some algorithms, so the information of one pixel in the plain-image can be spread into the entire cipher image [23,35,36]. In some other image encryption systems [12,23,35,36,37,38,39], the key streams for encryption are related to the plain images. For instance, in [38], the initial state conditions of chaotic maps are extremely dependent on plain image, so the generated key streams are highly sensitive to the original pictures to resist known/chosen plaintext attacks. In [39], Liu et al. presented a fast image encryption algorithm. In the scheme, the iteration values of 2D-SIMM are influenced by the encrypted pixel value, and the step size of cyclic shift and the secret key for substitution will be different with different images. Therefore, the designed algorithm can resist known-plaintext and chosen-plaintext attacks. A chaos-based color image encryption algorithm was proposed in [41], in which the color image is converted into three bit-level images and combined to one bit-level image. Then, only permutation operation is performed to encrypt the integrated bit-level image to reduce the execution time. Some of the plaintext-related algorithms mentioned above present low space keys, high encryption time, or insufficient security to resist powerful known/chosen plaintext attack. For instance, the encryption algorithm presented in [34] was cryptanalyzed and broken with chosen-plaintext attack in [42].

Based on the analysis above, this paper presents a new symmetric color image encryption system based on a plaintext-related random access bit-permutation mechanism (PRRABPM). Our encryption system has the following features:

(1) For color image encryption, bit-level permutation-based encryption algorithms have the advantages that they can achieve the interaction between RGB components in the scrambling phase, which can improve the security of encryption. So, this paper proposes a new random access bit-permutation mechanism in the permutation stage, which can obtain a good permutation effect and mask the statistical properties of the original image even though the diffusion key or diffusion sequence is cracked.
(2) In order to obtain high plain sensitivity and key sensitivity, the key streams used in random access bit-permutation mechanism operation are extremely dependent on plain image in an ingenious way. Therefore, the encryption system is sensitive to tiny differences in key and original images, which means that it can efficiently resist chosen-plaintext and chosen-ciphertext attacks.
(3) Not only color images but also gray images of any size can be encrypted by our encryption scheme.
(4) For the excellent performance of PRRABPM used in the permutation stage, the permutation-diffusion operation in our encryption scheme is performed only once.

The structure of this paper is as follows. Section 2 briefly reviews the chaotic maps used in this dissertation: tent map, Chebyshev map, and piecewise linear map. Section 3 proposes a plaintext-related random access bit permutation mechanism (PRRABPM). In Section 4, we evaluate the performance of the new algorithm and show the results of simulation and analysis. The last section gives a conclusion.

## 2. The Involved Chaotic Systems

One-dimensional chaotic system which has the advantages of simple structure and easy realization is an ideal choice for fast encryption of large-capacity data. In this section, three 1D chaotic maps for our new chaotic encryption scheme are briefly discussed: tent map, Chebyshev map, and piecewise linear map.

### 2.1. Tent Map

A chaotic tent map (CTM) is a piecewise linear map which can be defined as:

(1) xn+1=F1(xn,u)=uxnuxn22, xn<0.5u(1−xn)u(1−xn)22, xn≥0.5,

where u∈(0,4] and $x_{n}\in \left(0,1\right)$ is the output chaotic sequence.

**Figure 1:**
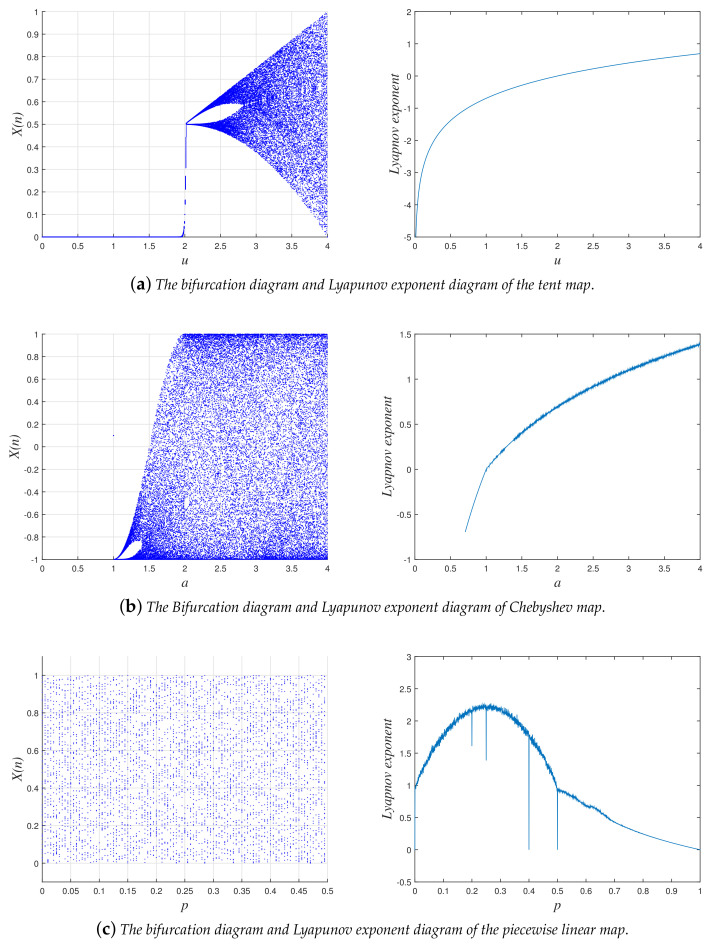
The bifurcation diagrams and Lyapunov exponent diagrams.

The chaotic sequence generated by the chaotic map is used for confusing and diffusing the pixels or bits of the original image. To a large extent, the uniform level of the output chaotic sequence determines the security of the encryption system. Bifurcation analysis and Lyapunov exponent analysis are often used to measure the chaotic property of the chaotic system. As shown in Figure 1a, the tent map has a chaotic behavior when parameter u∈(2,4].

### 2.2. Chebyshev Map

The Chebyshev map has similar chaotic behavior to the tent map. The expression of Chebyshev maps is shown as:

(2) $$x_{n+1}=F_{2}\left(x_{n},a\right)=\cos\left(a\times arc\cos x_{n}\right),$$

where $x_{n}\in \left[-1,1\right]$ is the output chaotic sequence and the a∈N parameter and $x_{0}$ is the initial value of the sequence which can be viewed as the secret key in our proposed algorithm. When a≥2, the bifurcation behavior of the Chebyshev system enters chaotic state and the Lyapunov exponent of Chebyshev maps is positive as shown in Figure 1b.

### 2.3. Piecewise Linear Map

The piecewise linear chaotic map (PWLCM) is a famous 1D chaotic map composed of multiple linear segments. The PWLCM is defined by the following equation:

(3) xn+1=F3(xn,p)=xnxnpp, 0<xn<p(xn−p)(xn−p)(0.5−p)(0.5−p), p<xn<0.5F(1−xn,p), 0.5<xn<1,

where $x_{n}\in \left(0,1\right)$ is the output chaotic sequence and *p* is the control parameter satisfying p∈(0,0.5).

The parameter *p* can serve as a key, as the PWLCM system is chaotic and has few periodic windows in its bifurcation diagram in the whole range of the parameter [43]. For a uniform invariant distribution and excellent ergodicity, the PWLCM chaotic map is employed in the proposed algorithm with a given initial value $x_{0}$ and control parameter *p*. The bifurcation diagram and Lyapunov Exponent diagram of PWLCM are shown in Figure 1c.

## 3. New Image Encryption Algorithm

**Figure 2:**
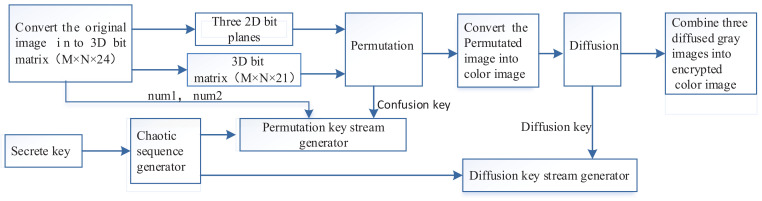
The proposed cryptosystem.

In this section, we propose a new image encryption algorithm. A block diagram of the proposed image encryption system is shown in Figure 2. Color images with RGB components can be viewed as a 3D matrix with size M×N×3. Each component in RGB components can be represented by an 8-bit binary. The value of the pixel at coordinate (x,y,z) is denoted as

(4) $$P\left(x,y,z\right)={\left\{R_{1}R_{2}\cdots R_{8}G_{1}G_{2}\cdots G_{8}B_{1}B_{2}\cdots B_{8}\right\}}_{\left(x,y,z\right)}.$$

**Figure 3:**
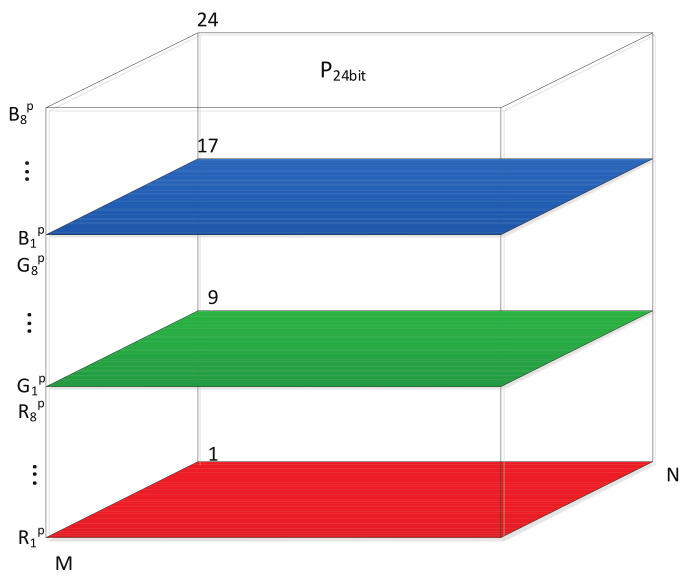
3D bit matrix of color images.

As shown in Figure 3, color images can be transformed into a 3D bit matrix (width, height, and bit-length). The binary bit-planes $R_{i}^{p}$, $G_{i}^{p}$, and $B_{i}^{p}\left(i=1,2,\cdots ,8\right)$ were transformed from the RGB components of the original color image. Since the bits in the higher bit-planes of the component contribute more effect to visualization, the lowest bit-plane of the RGB component of the 3D matrix $R_{1}^{p},G_{1}^{p},B_{1}^{p}$ is picked up to permute independently. The remaining bit-planes of each RGB component are used to form a new 3D matrix with size M×N×21, denoted as $P_{21bit}$ .The new formed matrix is permuted with PRRABPM.

### 3.1. Permutation Stage of the Encryption System Using PRRABPM

**Figure 4:**
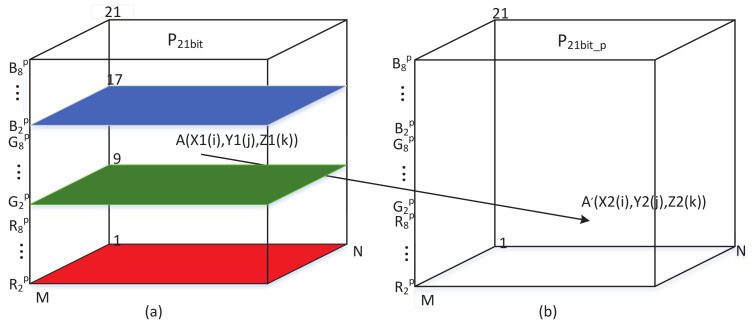
Permutation with the plaintext-related random access bit-permutation mechanism (PRRABPM). (**a**) The remaining bit-planes are used to form $P_{21bit}$; (**b**) Three sequences are mapped to the width, height, and bit-length of the permuted bit matrix $P_{21bit\_p}$.

Step 1: Decompose the M×N original color image into 24 bit-planes with size M×N. As illustrated in Figure 3, the 24 bit-planes are transformed into a 3D bit matrix, denoted as $P_{24bit}$. The lowest bit-plane $\left(R_{1}^{p},G_{1}^{p},B_{1}^{p}\right)$ of RGB components are picked up from the 3D matrix $P_{24bit}$. The remaining bit-planes are used to form $P_{21bit}$, as illustrated in Figure 4a.

Step 2: The sequences X1, Y1, and Z1 used in PRRABPM are obtained in steps 2 and 3 by sorting and searching operations. Iterate the tent map in Equation (1), the Chebyshev map in Equation (2), and PWLCM in Equation (3) $\left(3M/2+N_{0}\right)$, $\left(3N/2+N_{0}\right)$, and $\left(45+N_{0}\right)$ times, respectively. The first $N_{0}$ elements are discarded to avoid the harmful effects. Then, three new sequences $X_{1},Y_{1},Z_{1}$ are obtained with sizes 3M/2, 3N/2, and 45, given as

(5) $$\left\{\begin{array}{c} X_{1}=\left\{x_{1,1}+x_{1,2}+x_{1,3}+\cdots +x_{1,3M/2}\right\},\\ Y_{1}=\left\{y_{1,1}+y_{1,2}+y_{1,3}+\cdots +y_{1,3N/2}\right\},\\ Z_{1}=\left\{z_{1,1}+z_{1,2}+z_{1,3}+\cdots +z_{1,45}\right\}. \end{array}\right.$$

$N_{0}$ is a constant and can serve as the security key. The parameter of the three chaotic maps are defined as u=3.999998, a=4, and p=0.256, respectively. Note that these three parameters are not used as security keys.

Step 3: Take the first M, N and 21 elements of the sequences $X_{1},Y_{1},Z_{1}$ to form three new sequences $X_{2},Y_{2},Z_{2}$, given by

(6) $$\left\{\begin{array}{c} X_{2}=\left\{x_{1,1}+x_{1,2}+x_{1,3}+\cdots +x_{1,M}\right\},\\ Y_{2}=\left\{y_{1,1}+y_{1,2}+y_{1,3}+\cdots +y_{1,N}\right\},\\ Z_{2}=\left\{z_{1,1}+z_{1,2}+z_{1,3}+\cdots +z_{1,21}\right\}. \end{array}\right.$$

Then, the three sequences are sorted by ascending order to obtain the sorted sequences $X_{2s},Y_{2s},Z_{2s}$. By using sequence $X_{2s}$ and the corresponding original sequence $X_{2}$, the sequence X1 mapping to the width of the matrix $P_{21bit}$ can be obtained. The specific approach is that if the *i*-th element value in $X_{2}$ is equal to the *j*-th element value in $X_{2s}$, the *i*-th element value in X1 is *j*. Similarly, by using sequences $Y_{2s}$, $Y_{2}$ and $Z_{2s}$, $Z_{2}$, the other two sequences Y1, Z1 mapping to the height and bit-length of the matrix can be obtained, respectively, given by

(7) $$\left\{\begin{array}{c} X1=\{x_{1}+x_{2}+x_{3}+\cdots +x_{M}\},\\ Y1=\{y_{1}+y_{2}+y_{3}+\cdots +y_{N}\},\\ Z1=\{z_{1}+z_{2}+z_{3}+\cdots +z_{21}\}. \end{array}\right.$$

Step 4: Obtain another three sequences X2, Y2, and Z2 with the sizes M, N, and 21 used in PRRABPM in step 4 by sorting and searching operations. The generation of the sequences X2 and Y2 is extremely dependent on plain image in an ingenious way. Three sequences are mapped to the width, height, and bit-length of the permuted bit matrix $P_{21bit\_p}$ which is illustrated in Figure 4b. The specific approach using the plaintext-related algorithm can be described as follows:

(1) Calculating the sum of the elements in $R_{1}^{p},G_{1}^{p},B_{1}^{p}$, and $P_{24bit}$, respectively, one can get
  (8) $$sum_{1}^{R}=\sum_{i=1}^{M}\sum_{j=1}^{N}R_{1}^{p}\left(i,j\right),$$
  (9) $$sum_{1}^{G}=\sum_{i=1}^{M}\sum_{j=1}^{N}G_{1}^{p}\left(i,j\right),$$
  (10) $$sum_{1}^{B}=\sum_{i=1}^{M}\sum_{j=1}^{N}B_{1}^{p}\left(i,j\right),$$
  (11) $$Sum=\sum_{i=1}^{M}\sum_{j=1}^{N}\sum_{k=1}^{24}P_{24bit}\left(i,j,k\right).$$
(2) Calculating the value of the parameters of num1 and num2, respectively, one can get
  (12) $$f1=\frac{10^{5}\times \left(sum_{1}^{R}+sum_{1}^{G}+sum_{1}^{B}\right)}{Sum},$$
  (13) $$num1=floor(\;\mathrm{mod}\;\left(\left(f1-floor\left(f1\right)\right)\times 10^{10},floor\left(0.5\times M\right)\right))+1,$$
  (14) $$num2=floor(\;\mathrm{mod}\;\left(\left(f1-floor\left(f1\right)\right)\times 10^{10},floor\left(0.5\times N\right)\right))+1.$$
  If all the values of $sum_{1}^{R}$, $sum_{1}^{G}$, and $sum_{1}^{B}$ are zero, the values of num1 and num2 are set to $\frac{M}{2}$ and $\frac{N}{2}$, respectively.
(3) Obtain the sequence $X_{3},Y_{3},Z_{3}$ with sizes M, N, and 21, given by:
  (15) $$\left\{\begin{array}{c} X_{3}=\left\{x_{1,num1}+x_{1,num1+1}+x_{1,num1+2}+\cdots +x_{1,num1+M-1}\right\},\\ Y_{3}=\left\{y_{1,num1}+y_{1,num1+1}+y_{1,num1+2}+\cdots +y_{1,num1+N-1}\right\},\\ Z_{3}=\left\{z_{1,22}+z_{1,23}+z_{1,24}+\cdots +z_{1,42}\right\}, \end{array}\right.$$
  and then, by way of Step 3, the sequences X2,Y2,Z2 can be obtained using the sequences $X_{3},Y_{3},Z_{3}$. One can get
  (16) $$\left\{\begin{array}{c} X2=\{x_{1}^{{}^{\prime }}+x_{2}^{{}^{\prime }}+x_{3}^{{}^{\prime }}+\cdots +x_{M}^{{}^{\prime }}\},\\ Y2=\{y_{1}^{{}^{\prime }}+y_{2}^{{}^{\prime }}+y_{3}^{{}^{\prime }}+\cdots +y_{N}^{{}^{\prime }}\},\\ Z2=\{z_{1}^{{}^{\prime }}+z_{2}^{{}^{\prime }}+z_{3}^{{}^{\prime }}+\cdots +z_{21}^{{}^{\prime }}\}. \end{array}\right.$$

Step 5: Permute the three 2D bit matrices $R_{1}^{p},G_{1}^{p},\mathrm{and}\;B_{1}^{p}$ independently. The permutation equations can be described as

(17) $$\left\{\begin{array}{c} R_{1}^{p^{\prime }}\left(X1\left(i\right),Y1\left(j\right)\right)=R_{1}^{p}\left(i,j\right),\\ G_{1}^{p^{\prime }}\left(X1\left(i\right),Y1\left(j\right)\right)=G_{1}^{p}\left(i,j\right),\\ B_{1}^{p^{\prime }}\left(X1\left(i\right),Y1\left(j\right)\right)=B_{1}^{p}\left(i,j\right), \end{array}\right.$$

where i=1,2,⋯,M;j=1,2,⋯,N and $R_{1}^{p^{\prime }},G_{1}^{p^{\prime }},B_{1}^{p^{\prime }}$ are the permuted bit matrices.

Step 6: Permute the 3D bit matrix $P_{21bit}$ using PRRABPM, given by

(18) $$P_{21bit\_p}\left(X2\left(i\right),Y2\left(j\right)Z2\left(k\right)\right)=P_{21bit}\left(X1\left(i\right),Y1\left(j\right)Z1\left(k\right)\right),$$

where i=1,2,⋯,M;j=1,2,⋯,N;k=1,2,⋯,21.

According to Equation (18) and Figure 4, the sequences X1,Y1,Z1 are used to access the bit element in $P_{21bit}$ randomly and the sequences X2,Y2,Z2 are used to permute the bit positions in $P_{21bit\_p}$.

Step 7: In order to obtain good permutation performance and make the RGB components of the color image interact with each other more sufficiently, the permutated 2D bit matrices $R_{1}^{p^{\prime }},G_{1}^{p^{\prime }},\mathrm{and}\;B_{1}^{p^{\prime }}$ are added to the permutated 3D bit matrix $P_{21bit\_p}$ to form the permutated 3D bit matrix $P_{24bit\_p}$ in the following way. Firstly, K1,K2,K3 are calculated using the value of $z_{1,43},z_{1,44},z_{1,45}$ in $Z_{2}$. One can get

(19) $$\left\{\begin{array}{c} K1=floor(z_{1,43}\times 10^{9}\;\mathrm{mod}\;7)+2,\\ K2=floor(z_{1,44}\times 10^{9}\;\mathrm{mod}\;7)+9,\\ K3=floor(z_{1,45}\times 10^{9}\;\mathrm{mod}\;7)+16. \end{array}\right.$$

The bit matrix $B_{1}^{p^{\prime }}$ is added to the bit-plane between the (K1−1)-th and the K1-th bit-plane of $P_{21bit\_p}$. Similarly, the bit matrix $R_{1}^{p^{\prime }}$ is added to the bit-plane between the (K2−1)-th and the K2-th bit-plane, and the bit matrix $G_{1}^{p^{\prime }}$ is added between the (K3−1)-th and the K3-th bit-planes.

### 3.2. Diffusion Stage of the Encryption System

Note that the diffusion operation is performed at the pixel-level.

Step 1: Firstly, the matrix $P_{24bit\_p}$ is converted into the color image $P_{p}$ with size M×N×3, and then the image $P_{p}$ can be divided into RGB components. Secondly, three gray images are transformed into 1D pixel arrays ($P_{R\_p},P_{G\_p},P_{B\_p}$), respectively. One can get

(20) $$\left\{\begin{array}{c} P_{R\_p}=\left\{{p_{R\_p}}_{1},{p_{R\_p}}_{2},{p_{R\_p}}_{3}\cdots {p_{R\_p}}_{\left(M\times N\right)}\right\},\\ P_{G\_p}=\left\{{p_{G\_p}}_{1},{p_{G\_p}}_{2},{p_{G\_p}}_{3}\cdots {p_{G\_p}}_{\left(M\times N\right)}\right\},\\ P_{B\_p}=\left\{{p_{B\_p}}_{1},{p_{B\_p}}_{2},{p_{B\_p}}_{3}\cdots {p_{B\_p}}_{\left(M\times N\right)}\right\}. \end{array}\right.$$

Step 2: Obtain the diffusion matrix $D=\{d_{1},d_{2},d_{3}\cdots d_{\left(M\times N\right)}\}$, given by

(21) $$D\left(k\right)=\;\mathrm{mod}\;(X1\left(i\right)\times 10^{13}+Y1\left(j\right)\times 10^{13},256),$$

where i=1,2,⋯,M;j=1,2,⋯,N;k=i×j.

Step 3: Obtain the encrypted image pixel arrays $C_{R},C_{G},C_{B}$ using the 1D pixel arrays $P_{R\_p},P_{G\_p},P_{B\_p}$ and the diffusion matrix *D*, given by

(22) $$\left\{\begin{array}{c} C_{R}\left(i\right)=\;\mathrm{mod}\;\left(P_{R\_p}\left(i\right)⊗D\left(i\right)+D\left(i\right),256\right)⊗C_{R}\left(i-1\right),\\ C_{G}\left(i\right)=\;\mathrm{mod}\;\left(P_{G\_p}\left(i\right)⊗D\left(i\right)+D\left(i\right),256\right)⊗C_{G}\left(i-1\right),\\ C_{B}\left(i\right)=\;\mathrm{mod}\;\left(P_{B\_p}\left(i\right)⊗D\left(i\right)+D\left(i\right),256\right)⊗C_{B}\left(i-1\right), \end{array}\right.$$

where i=1,2,⋯,M×N, and symbol “⊗” represents bitwise exclusive or operator.

Step 4: Treat the encrypted image pixel arrays $C_{R},C_{G},C_{B}$ as RGB components of a color image so that an encrypted color image with size M×N×3 can be obtained.

### 3.3. Decryption Process

**Figure 5:**
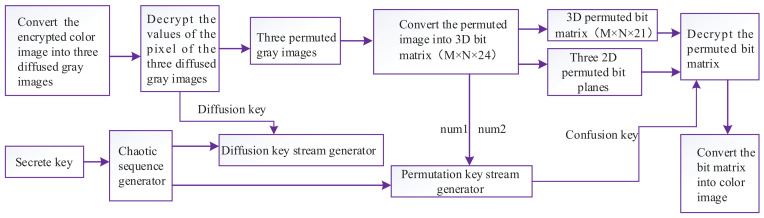
Flowchart of the decryption process.

The decryption procedure is the inverse process of encryption. The flowchart of the decryption process is shown in Figure 5. The diffusion equation used in decryption is given as

(23) $$\left\{\begin{array}{c} P_{R\_p}\left(i\right)=\;\mathrm{mod}\;\left(C_{R}\left(i\right)⊗C_{R}\left(i-1\right)+256-D\left(i\right),256\right)⊗D\left(i\right),\\ P_{G\_p}\left(i\right)=\;\mathrm{mod}\;\left(C_{G}\left(i\right)⊗C_{G}\left(i-1\right)+256-D\left(i\right),256\right)⊗D\left(i\right),\\ P_{B\_p}\left(i\right)=\;\mathrm{mod}\;\left(C_{B}\left(i\right)⊗C_{B}\left(i-1\right)+256-D\left(i\right),256\right)⊗D\left(i\right), \end{array}\right.$$

where $P_{R\_p},P_{G\_p},P_{B\_p}$ are 1D pixel arrays.

(24) $$A\left(i,j\right)=A_{p}\left(X1\left(i\right),Y1\left(j\right)\right),$$

where *A* is the 2D bit matrix which is permuted independently in the encryption process and $A_{p}$ is the corresponding permuted 2D bit matrix.

The permutation equation used in decryption is given as

(25) $$P_{21bit}\left(X1\left(i\right),Y1\left(j\right),Z1\left(k\right)\right)=P_{21bit\_p}\left(X2\left(i\right),Y2\left(j\right),Z2\left(k\right)\right)$$

where $P_{21bit}$ is the 3D bit matrix which is permuted using PRRABPM in the encryption process and $P_{21bit\_p}$ is the corresponding permuted 3D bit matrix.

### 3.4. Simulation Results

**Figure 6:**
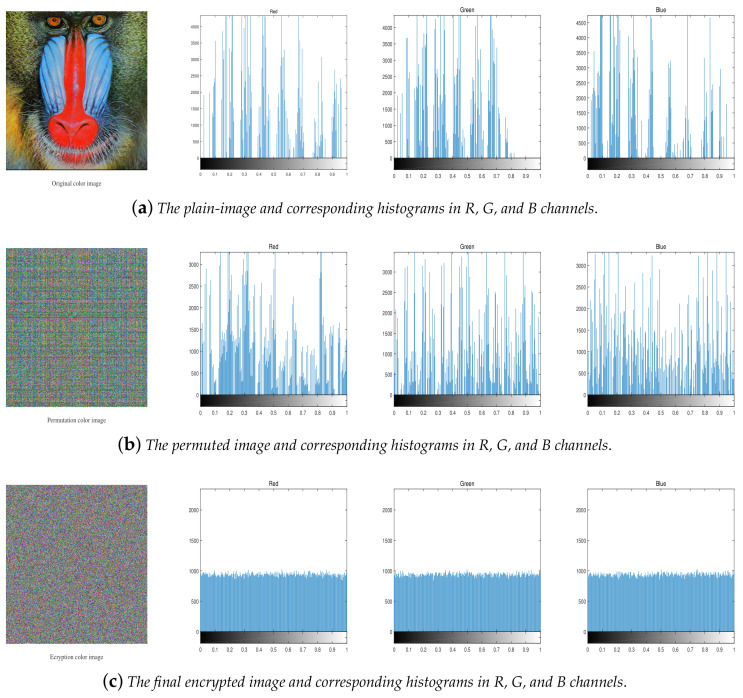
The histograms of plain-image, permuted image, and final encrypted images.

In this section, we evaluate the performance of the proposed scheme. The initial values of the tent map, the Chebyshev map, and the piecewise linear map are chosen as $key_{\_x0}=0.22521231547896$; $key_{\_y0}=0.58749654123587$; and $key_{\_z0}=0.98564123475621$, respectively, and $N_{0}=2000$. A baboon is used as the testing plain-image. The plain-image, the results of encryption-decryption images, and their corresponding distribution histograms are shown in Figure 6.

## 4. Security Analysis

### 4.1. Security Key Space

For a security encryption algorithm, its key space should be larger than $2^{100}$ [44]. There are four secret keys in the proposed encryption algorithm, including the initial values $\left(key_{\_x0},key_{\_y0},key_{\_z0}\right)$ of the three chaotic maps and the iteration times $N_{0}$. For these four secret keys, $key_{\_x0}$ belongs to (0,1), $key_{\_y0}$ belongs to (−1,1), $key_{\_z0}$ belongs to (0,1), and $N_{0}$ belongs to (1000,2500]. As the computational precision of double-precision numbers is taken as $10^{16}$, the key space is $key_{total}=10^{16}\times 10^{16}\times 10^{16}\times 1500\approx 2^{170}$. So, the key space of the proposed cryptosystem is large enough to resist brute force attack. In Table 2, the comparison between our method and similar image encryption algorithms is given, showing that the key space size of the proposed algorithm is larger than most of the similar algorithms.

### 4.2. Statistical Analysis

#### 4.2.1. Histogram Analysis

The histogram of the encrypted image is often used to measure the security of the encryption system. For a secure encryption system, the histogram of the encrypted image should be flat, which can resist statistical attacks. The histograms of the plain-images and the corresponding cipher-images are shown in Figure 6a,c. As shown in Figure 6c, the encrypted image is completely scrambled and its histogram has a good uniform distribution, so it can resist statistical attacks. Furthermore, the histogram of the permuted image shown in Figure 6b is different from the histogram of the original image to some extent, so even though the diffusion key or diffusion sequence is cracked, the statistical information of the original image can be masked.

#### 4.2.2. Correlation Analysis

The correlation coefficient of the pixels of the plain-image is always high because the adjacent pixels in the plain-image have a high correlation which can be used by attackers. So, the correlation coefficients of the pixels should be significantly reduced after the plain-image is encrypted. According to Equation (26), we calculate the correlation coefficients of the four directions, including the vertical, horizontal, diagonal, and anti-diagonal directions.

(26) $$r_{xy}=\frac{\mathrm{cov}(x,y)}{\sqrt{D(x)}\times \sqrt{D(y)}},$$

where $\mathrm{cov}\left(x,y\right)=\frac{1}{N}\sum_{i=0}^{N}(x_{i}-E\left(x\right))\left(y_{i}-E\left(y\right)\right)$, $D\left(x\right)=\frac{1}{N}\sum_{i=0}^{N}(x_{i}-E\left(x\right){)}^{2}$, E(x) $=\frac{1}{N}\sum_{i=0}^{N}x_{i}$. x,y are two adjacent pixel values from four directions as mentioned above, and *N* is the number of image pixels.

The correlation coefficients of plain-images and the corresponding cipher-images are provided in Table 3, including the vertical (V), horizontal (H), diagonal (D), and anti-diagonal (A) directions. As shown in Table 3, the correlation coefficients of the plain-images are close to 1, but the correlation coefficients of the cipher-images are close to 0. This indicates that the proposed algorithm can resist a statistical attack. Detailed results compared with some related references are given in Table 4.

**Figure 7:**
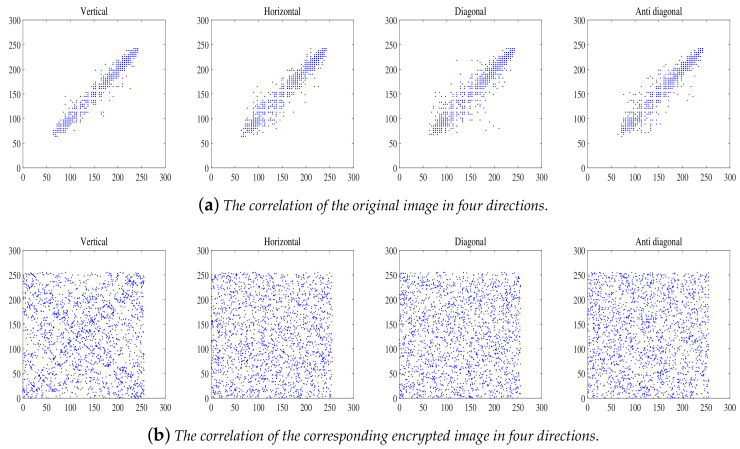
Correlation analysis of the original image and the corresponding encrypted image in R channel (The original image is Lena).

In order to evaluate the correlation property of images, we randomly select 2000 pixels from the plain-image or their corresponding cipher-image. The correlation diagram among adjacent pixels at vertical, horizontal, diagonal, and anti-diagonal directions of the R channel are shown in Figure 7. As shown in Figure 7, the values of the adjacent pixels of the cipher-image are completely different.

#### 4.2.3. Key Sensitivity and Plaintext Sensitivity Analysis

A differential attack is usually used to break a cryptosystem. For a secure encryption system to effectively resist such an attack, it should be sensitive to any tiny modification in the keys or the original image. The NPCR (number of pixels change rate) and UACI (unified average changing intensity) are usually used to evaluate the sensitivity of the key and plain-image. NPCR and UACI are expressed in the following equation:

(27) $$\left\{\begin{array}{c} NPCR=\sum_{i=0}^{H}\sum_{j=0}^{W}D(i,j)\times 100\%,\\ UACI=\frac{1}{W\times H}\sum_{i=0}^{H}\sum_{j=0}^{W}\frac{\left|c_{1}\left(i,j\right)-c_{2}\left(i,j\right)\right|}{255}\times 100\%, \end{array}\right.$$

where $c_{1},c_{2}$ are encrypted images, and $D\left(i,j\right)=\left\{\begin{array}{c} 0,\;if\;c_{1}\left(i,j\right)=c_{2}\left(i,j\right),\\ 1,\;if\;c_{1}\left(i,j\right)\neq c_{2}\left(i,j\right). \end{array}\right.$

In this simulation, four plain-images were used to evaluate the key sensitivity. The proposed algorithm has four secret keys $\left(key_{\_x0},key_{\_y0},key_{\_z0},N_{0}\right)$. For example, the sensitivity of $key_{x0}$ is evaluated here. First, we selected 200 key groups $k_{ey}\left(i\right)\;=\;\left(key_{\_x0}\left(i\right),key_{\_y0}\left(i\right),key_{\_z0}\left(i\right),N_{0}\left(i\right)\right)\left(i=1,2,3\cdots 200\right)$ from the security key space randomly and then we used every key group to encrypt the plain-images. Then, the corresponding cipher-images $C_{1}\left(i\right)\left(i=1,2,3\cdots 200\right)$ could be obtained. Secondly, a slight change $10^{-15}$ was added into the secret key $key_{\_x0}\left(i\right)$ of the key group. The values of the remaining three keys $\left(key_{\_y0}\left(i\right),key_{\_z0}\left(i\right),N_{0}\left(i\right)\right)$ were unchanged. Then, the key group containing the modified key was used to encrypt the plain-images again to obtain corresponding cipher-images $C_{2}\left(i\right)\left(i=1,2,3\cdots 200\right)$. According to Equation (27), 200 pairs of NPCR and UACI could be calculated. The average values of NPCR and UACI are shown in Table 5. It should be noted that the key sensitivity of $key_{\_y0},key_{\_z0},N_{0}$ was evaluated in the same way.

As shown in Table 5, the NPCR and UACI values were very close to the ideal values(NPCR:99.6094 and UACI:33.4635), indicating that the encryption system is sensitive to any tiny modifications in keys.

**Figure 8:**
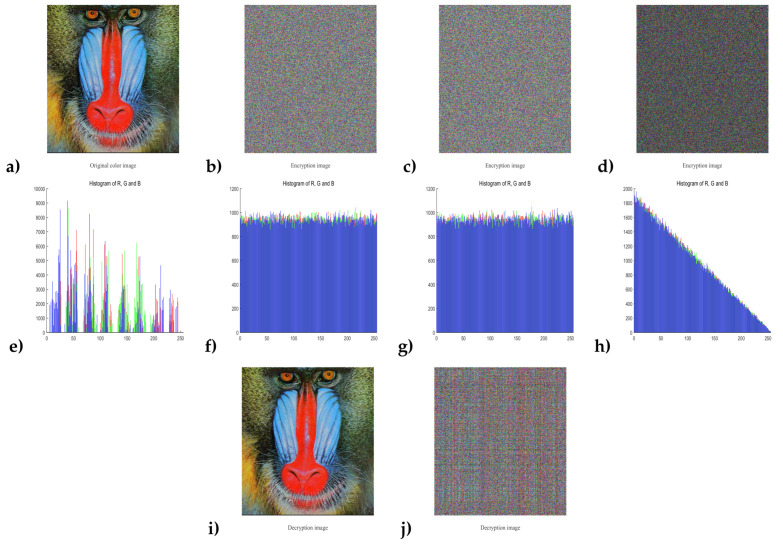
The key sensitivity test: (**a**,**e**): the original image and corresponding histogram; (**b**,**f**): the encrypted image E1 with the security key set $k_{ey}\left(1\right)$ and corresponding histogram; (**c**,**g**): the encrypted image E2 with the security key set $k_{ey}\left(2\right)$ and corresponding histogram; (**d**,**h**): the pixel-by-pixel difference |E1−E2| and corresponding histogram; (**i**): the decrypted image from E1 using the correct security key set $k_{ey}\left(1\right)$ ; (**j**): the decrypted image from E2 using an incorrect security key set $k_{ey}\left(1\right)$.

Another key sensitivity test is shown in Figure 8. Firstly, a key set was chosen from the security key space denoted as $k_{ey}\left(1\right)=\left(0.2252123154789600,0.5874965412358700,0.9856412347562100,2000\right)$. To evaluate the sensitivity of $key_{\_x0}$, a slight change $10^{-16}$ is added into the secret key $key_{\_x0}\left(1\right)$ while the others were unchanged. Then the key denoted as $k_{ey}\left(2\right)=(0.2252123154789601,$ 0.5874965412358700,0.9856412347562100,2000) could be obtained. After that, we used $k_{ey}\left(1\right)$ and $k_{ey}\left(2\right)$ to encrypt the same original image as shown in Figure 8a to obtain two corresponding cipher-images denoted as E1 and E2, shown in Figure 8b,c. The image of pixel-to-pixel difference |E1−E2| is shown in Figure 8d, and its histogram is shown in Figure 8h, which can prove that a tiny change $10^{-16}$ in the security key will result in a significant change in cipher-image. Finally, we used $k_{ey}\left(1\right)$ to decrypt the cipher images E1 and E2 individually, and the decryption results are shown in Figure 8i,j. As can be seen, only the correct key can completely reconstruct the original image. Thus, the proposed algorithm has high key sensitivity.

In order to evaluate the sensitivity to small changes in the plain-image, we selected 200 key groups $k_{ey}\left(i\right)=\left(key_{\_x0}\left(i\right),key_{\_y0}\left(i\right),key_{\_z0}\left(i\right),N_{0}\left(i\right)\right)\left(i=1,2,3\cdots 200\right)$ from the security key space and selected 200 pixels denoted as $P_{ixel\_i}\left(x,y,z\right)\left(i=1,2,3\cdots 200\right)$ from the original color images at random location (x,y,z). For instance, the cipher image $C_{1}^{{}^{\prime }}$ can be obtained by using key $k_{ey}\left(1\right)$ to encrypt the plain-image. Then, we modified the value of pixel $P_{ixel\_1}\left(x,y,z\right)$ in the plain-image slightly, as shown in Equation (28).

(28) $$P_{ixel}\left(x_{i},y_{j},z_{k}\right)=\;\mathrm{mod}\;\left(P_{ixel}\left(x_{i},y_{j},z_{k}\right)+1,256\right).$$

The plain-image containing the modified pixel $P_{ixel\_1}\left(x,y,z\right)$ was encrypted with the same key $k_{ey}\left(1\right)$, so that the corresponding cipher image $C_{2}^{{}^{\prime }}$ could be obtained. According to Equation (27), NPCR and UACI were calculated using $C_{1}^{{}^{\prime }}$ and $C_{2}^{{}^{\prime }}$. Finally, by using different keys in key groups and slightly changed plain-images, a total of 200 pairs of NPCR and UACI were calculated. The average of the NPCR and UACI is shown in Table 6. Through Table 6, we can see that the NPCR and UACI values were very close to the ideal values, indicating that the encryption system is sensitive to any little change in plain-image.As shown in Table 7 and Table 8, our NPCR and UACI mean values passed the randomness test, compared with the expected values [45].

#### 4.2.4. Information Entropy Analysis

Information entropy is an important performance index which can be used to measure the randomness and unpredictability of an information source. So, it is usually used to measure the strength of a cryptosystem, given by

(29) $$H\left(m\right)=\sum_{i=0}^{2^{N}-1}p(m_{i})\log\frac{1}{p(m_{i})},$$

where *m* denotes an information source, and $2^{N}$ denotes the number of all possible pixel values. If *m* has $2^{N}$ possible values, the corresponding theoretical value should be H(m)=N. So, for a cipher-image with 256 gray levels, the ideal value for the information entropy is 8. According to Equation (29), the information entropy of seven plain-images and their corresponding cipher-images—which were encrypted by our algorithm and other recent algorithms in the literature—were calculated as shown in Table 9. From this table, we can see that the information entropies of the cipher-images (RGB components) were all close to the ideal value. In other words, the cipher-images had a good property of randomness and the unpredictability.

#### 4.2.5. Encrypted Time Analysis

In practice, quick running speed is also significant for a good cryptosystem. The experimental environment was MATLAB R2014b with Intel Core i7-7500U CPU@ 3.5 GHz and 4.0 GB RAM on Windows 10 OS. Because the proposed scheme is bit-level permutation-based and related to plain images, encrypted time analysis was performed in comparison with similar algorithms for references [25,37,40]. Murillo-Escobar et al. presented a plaintext-related color image encryption algorithm based on total plain image characteristics and chaos in [37], in which the chaotic sequence used in permutation and diffusion is related to the total plain image characteristics. Then, the Z value needs to be inserted into the encrypted image, as it cannot be calculated from the encrypted image during the decryption process. In [40], a novel image encryption algorithm is proposed, in which the plain image is permuted by using the 2D rectangular transform. Then, in the diffusion stage, the previous encrypted pixel is used to encrypt the current pixel, while the first encrypted pixel in the chipper is related to all the pixels in the plain image. In [25], Xu et al. presented a novel bit-level image encryption algorithm that is based on piecewise linear chaotic maps (PWLCM). In the diffusion phase, the cycle shift operation is controlled by the sum of the binary sequence transformed from the plain image. In the confusion phase, the initial value of the chaotic map is determined by the permuted binary sequence.

The execution times for the proposed and comparable schemes including the time consumption in all of the encryption operations and key-stream generations are listed in Table 10. Encryption throughput (ET) in megabytes per second (MBps) and the number of cycles needed to encrypt one byte are also used to evaluate cryptosystem performance, and are given by Equations (30) and (31):

(30) $$ET=\frac{{\mathrm{Image}}_{Size}\left(Byte\right)}{Encryption_{Time}\left(second\right)},$$

(31) $$Number\;of\;cycles\;per\;Byte=\frac{CPU\;Speed_{\left(Hertz\right)}}{ET_{\left(Byte\right)}}.$$

Table 11 presents a comparison of ET and number of cycles needed to encrypt performance of the proposed cryptosystem with some recent cryptosystems. As shown in Table 10 and Table 11, the execution speed of the proposed scheme was slower than Murillo-Escobar’s algorithm, but more efficient than algorithms in [25,40]. There are three reasons for the relatively slower encryption speed compared to Murillo-Escobar’s algorithm. (1) Since the original color image has to be decomposed into 24 bit-planes in the permutation stage and then the permuted bit-planes are transformed into a color image, it will take more time than pixel-level permutation-based algorithms like [37]; (2) The image data to be processed in the permutation stage of bit-level permutation-based algorithms is eight times compared with pixel-level permutation-based algorithms; (3) The use of the sorting and searching operations in key-stream generation in our algorithms are particularly time-consuming. Considering its high level of security, the running speed is acceptable.

## 5. Conclusions

In contrast with the similar studies, a plaintext-related random access bit-permutation mechanism (PRRABPM) is presented in this paper. This method is used in the permutation stage to shuffle the RGB components of a color image at the same time, making these three components interact with each other. Furthermore, the key streams used in random access bit-permutation mechanism operation is extremely dependent on plain image in an ingenious way, which makes the encryption system sensitive to key and original images. Thus, the proposed encryption system can efficiently resist the chosen-plaintext and chosen-ciphertext attacks. Experimental results analysis including key space, histogram, correlation, sensitivity, information entropy, and speed are also given, showing that the proposed algorithm has a good security performance.

The proposed method may be used for image security communication applications. According to simulation results, the proposed algorithm is not suitable for real-time applications. Now, our main concern is the strength of the encryption algorithm and its ability to work with the limitations of security communication systems, which require further study. In our future work, we will consider this part in detail and improve the encryption speed. It may also be considered to integrate the image encryption with an image compression algorithm so as to enhance security for image transmission over communication systems.

## Figures and Tables

**Table 1:** Some approaches to the cryptanalysis of permutation-diffusion structure-based image ciphers.

Schemes	Cryptanalyzed by	Attacks Employed
Gao et al. (2008) [4]	Rhouma et al. (2014) [27]	chosen plaintext and ciphertext
Mirzaei et al. (2012) [5]	Wang et al. (2013) [28]	chosen-plaintext
Parvin et al. (2016) [7]	Norouzi et al. (2016) [29]	chosen-plaintext
Wang et al. (2012) [10]	Li et al. (2012) [30]	chosen-plaintext
Pak et al. (2017) [17]	Wang et al. (2018) [31]	chosen-plaintext
Zhu et al. (2011) [19]	Zhang et al. (2014) [32]	chosen plaintext and ciphertext
Zhang et al. (2016) [26]	Wu et al. (2018) [33]	chosen-plaintext

**Table 2:** Keyspace comparisons.

Schemes	Proposed Scheme	Ref. [17]	Ref. [23]	Ref. [34]	Ref. [35]	Ref. [37]	Ref. [38]
**Key space size**	$2^{170}$	$2^{138}$	$2^{241}$	$2^{104}$	$2^{128}$	$2^{128}$	$2^{572}$

**Table 3:** Correlation coefficients of some original images and the corresponding cipher-images in R, G, and B channels.

Image	Plain-Image					Cipher-Image			
		V	H	D	A	V	H	D	A
Lena	R	0.9753	0.9853	0.9734	0.9648	−0.0028	0.0046	0.0013	−0.0001
	G	0.9666	0.9802	0.9630	0.9536	0.0004	−0.0009	0.0007	0.0008
	B	0.9334	0.9558	0.9264	0.9198	−0.0029	−0.0007	−0.0050	0.0013
Baboon	R	0.9235	0.8740	0.8649	0.8670	0.0015	0.0002	−0.0014	0.0006
	G	0.8668	0.7759	0.7432	0.7494	0.0033	0.0018	0.0003	−0.0003
	B	0.9067	0.8844	0.8544	0.8540	0.0006	0.0004	0.0008	0.0012
Fruits	R	0.9936	0.9928	0.9897	0.9868	−0.0009	0.0003	−0.0002	0.0006
	G	0.9855	0.9848	0.9783	0.9694	−0.0019	−0.0004	0.0003	0.0010
	B	0.9265	0.9192	0.8809	0.8531	−0.0024	−0.0008	−0.0043	0.0015
Flowers	R	0.9718	0.9719	0.9504	0.9551	−0.0043	−0.0028	−0.0049	0.0045
	G	0.9510	0.9497	0.9123	0.9218	−0.0043	−0.0054	0.0017	0.0054
	B	0.9527	0.9527	0.9178	0.9256	−0.0035	0.0005	−0.0062	−0.0001

**Table 4:** Correlation coefficients of encrypted Lena image in R channel with different algorithms.

Direction	Original Image	Proposed Scheme	Ref. [15]	Ref. [37]	Ref. [17]	Ref. [38]	Ref. [40]
**Horizontal**	0.9853	0.0046	0.0027	0.0012	−0.0026	−0.0030	0.0005
**Vertical**	0.9753	−0.0028	−0.0013	0.0035	−0.0038	0.0025	−0.0070
**Diagonal**	0.9734	0.0014	0.0039	0.0056	0.0017	−0.0001	0.0006

**Table 5:** The mean NPCR (number of pixels change rate) and UACI (unified average changing intensity) of some encrypted images.

Image	NPCR (99.6094)				UACI (33.4635)		
		R	G	B	R	G	B
Lena	$key_{\_x0}$	99.5952	99.5957	99.5940	33.4657	33.4633	33.4666
	$key_{\_y0}$	99.6092	99.6079	99.6103	33.4623	33.4599	33.4582
	$key_{\_z0}$	99.5984	99.5883	99.5958	33.4637	33.4635	33.4665
	$N_{0}$	99.6088	99.6110	99.6098	33.4695	33.4600	33.4701
Baboon	$key_{\_x0}$	99.6077	99.6075	99.6083	33.4685	33.4635	33.4611
	$key_{\_y0}$	99.6091	99.6087	99.6110	33.4631	33.4656	33.4721
	$key_{\_z0}$	99.5977	99.5880	99.5965	33.4602	33.4696	33.4669
	$N_{0}$	99.6092	99.6112	99.6083	33.4678	33.4680	33.4637
Fruits	$key_{\_x0}$	99.6054	99.6065	99.6060	33.4639	33.4632	33.4634
	$key_{\_y0}$	99.6109	99.6086	99.6108	33.4665	33.4655	33.4641
	$key_{\_z0}$	99.5964	99.5884	99.5957	33.4629	33.4624	33.4685
	$N_{0}$	99.6101	99.6099	99.6093	33.4676	33.4637	33.4661
Flowers	$key_{\_x0}$	99.6026	99.6004	99.6018	33.4635	33.4535	33.4662
	$key_{\_y0}$	99.6076	99.6101	99.6071	33.4628	33.4641	33.4627
	$key_{\_z0}$	99.5977	99.5885	99.5937	33.4648	33.4696	33.4725
	$N_{0}$	99.6089	99.6084	99.6106	33.4687	33.4664	33.4633

**Table 6:** The mean NPCR and UACI of the some encrypted images, evaluating the plain-image sensitivity.

Image	NPCR (99.6094)			UACI (33.4635)		
	R	G	B	R	G	B
Lena	99.6086	99.6083	99.6104	33.4709	33.4683	33.4682
Baboon	99.6088	99.6099	99.6088	33.4548	33.4618	33.4684
Fruits	99.6091	99.6083	99.6091	33.4654	33.4647	33.4577
Flowers	99.6094	99.6096	99.6083	33.4681	33.4591	33.4663
Girl	99.6090	99.6101	99.6095	33.4670	33.4651	33.4641
Flower	99.6095	99.6084	99.6102	33.4629	33.4614	33.4615
Yacht	99.6086	99.6095	99.6087	33.4629	33.4654	33.4626
Lena in Ref. [15]	99.9985	99.9985	99.9985	–	–	–
Lena in Ref. [16]	99.6097	99.5994	99.5975	33.4476	33.4655	33.4769
Lena in Ref. [37]	99.4800	99.5158	99.4788	33.6322	33.7336	33.6005
Lena in Ref. [41]	99.6429	99.6140	99.6277	33.3935	33.5637	33.4814

**Table 7:** NPCR randomness test.

Tested Image Size 512 by 512	Theoretical NPCR Critical Value [45]		
	$N_{0.05}^{*}=99.5893$	$N_{0.01}^{*}=99.5810$	$N_{0.001}^{*}=99.5717$
Our mean NPCR Value	NPCR Test Results		
	0.05-level	0.01-level	0.001-level
99.6091	Pass	Pass	Pass

**Table 8:** UACI randomness test.

Tested Image Size 512 by 512	Theoretical UACI Critical Value [45]		
	$u_{0.05}^{*-}=33.3730$	$u_{0.01}^{*-}=33.3445$	$u_{0.001}^{*-}=33.3115$
	$u_{0.05}^{*+}=33.5541$	$u_{0.01}^{*+}=33.5826$	$u_{0.001}^{*+}=33.6156$
Our mean UACI Value	UACI Test Results		
	0.05-level	0.01-level	0.001-level
33.4691	Pass	Pass	Pass

**Table 9:** The result of the information entropy of some encrypted images.

Image	Plain-Image			Cipher-Image		
	R	G	B	R	G	B
Lena	5.0465	5.4576	4.8001	7.9992	7.9993	7.9994
Baboon	6.4998	6.4445	6.2709	7.9991	7.9992	7.9992
Fruits	7.5172	7.3230	6.7785	7.9992	7.9992	7.9993
Flowers	7.3824	7.2345	7.3641	7.9990	7.9990	7.9991
Girl	7.4346	7.2354	7.0578	7.9995	7.9995	7.9996
Flower	7.4428	7.4062	7.3371	7.9992	7.9993	7.9992
Yacht	7.6071	7.4062	7.3371	7.9994	7.9991	7.9993
Lena in Ref. [14]	–	–	–	7.9994	7.9994	7.9994
Lena in Ref. [16]	–	–	–	7.9914	7.9915	7.9916
Lena in Ref. [37]	–	–	–	7.9974	7.9975	7.9969
Lena in Ref. [38]	–	–	–	7.9972	7.9972	7.9976
Lena in Ref. [39]	–	–	–	7.9975	7.9972	7.9973
Lena in Ref. [41]	–	–	–	7.9942	7.9943	7.9942

**Table 10:** Encryption time (seconds).

Scheme	Color (512×512)	Color (256×256)	Gray (512×512)	Gray (256×256)	Platform
**Proposed**	4.6058	1.1347	1.8112	0.4389	Matlab
**Ref. [37] (2015)**	0.3722	0.1225	–	–	Matlab
**Ref. [40] (2017)**	14.8119	3.6175	–	–	Matlab
**Ref. [25] (2016)**	–	–	12.6917	3.1342	Matlab
**Ref. [46] (2013)**	–	–	0.030	0.0075	VisualC++
**Ref. [47] (2011)**	–	–	0.033	0.0078	C
**Ref. [16] (2016)**	0.009	0.002	–	–	C
**Ref. [48] (2013)**	–	–	0.92	0.16	Matlab

**Table 11:** Encryption throughput (ET) and number of cycles for one encrypted byte.

Algorithm	ET in MBps	Number of Cycles Per Byte	Platform
**Proposed**	0.165	20229.45	Matlab
**Ref. [37] (Murillo-Escobar et al., 2015)**	1.531	2180.18	Matlab
**Ref. [40] (Wu et al., 2017)**	0.052	64189.61	Matlab
**Ref. [25] (Xu et al., 2016)**	0.020	166893.006	Matlab
**Ref. [46] (Zhang et al., 2013)**	8.33	366.35	VisualC++
**Ref. [47] (Wang et al., 2011]**	8.01	369.08	C
**Ref. [16] (M. Farajallah et al., 2016)**	93.82	31.51	C
**Ref. [48] (Pareek et al., 2013)**	0.27	10596.38	Matlab
